# Biallelic variants in the *UTRN* gene cause a novel form of multiple congenital arthrogryposis

**DOI:** 10.3389/fgene.2025.1664424

**Published:** 2025-10-30

**Authors:** Evgeniya Melnik, Daria Akimova, Tatiana Markova, Eugene Tatarskiy, Anna Tvorogova, Viktoria Zabnenkova, Vladimir Kenis, Olga Agranovich, Mikhail Skoblov, Elena Dadali

**Affiliations:** ^1^ Research Centre for Medical Genetics, Moscow, Russia; ^2^ Center for Precision Genome Editing and Genetic Technologies for Biomedicine, Institute of Gene Biology, Russian Academy of Sciences, Moscow, Russia; ^3^ H. Turner National Medical Research Center for Children’s Orthopedics and Trauma Surgery of the Ministry of Health of the Russian Federation, Saint Petersburg, Russia

**Keywords:** UTRN, utrophin, arthrogryposis multiplex congenita, AMC, novel phenotype

## Abstract

Arthrogryposis multiplex congenita (AMC) is a large group of congenital conditions characterized by joint contractures affecting two or more body areas. A part of AMC type is caused by heterozygous pathogenic variants in genes encoding sarcomeric components of skeletal muscle fibers. Here we report a 7-year-old boy with a phenotype including AMC with dysmorphic facial features, short stature, congenital malformations of brain, colon and lacrimal canal. Trio whole-genome sequencing identified compound heterozygosity in the *UTRN* gene, consisting of a splicing variant in intron 57 (c.8434 + 1G>A) and a large heterozygous deletion spanning exons 3–51 (NM_007124.3). It is known that utrophin, the product of the *UTRN* gene, is an autosomal homologue and a fetal form of a protein of skeletal muscles - dystrophin. The presence of multiple malformations in the patient’s phenotype is consistent with ubiquitous expression of utrophin in the embryonic period. The RNA-seq analysis revealed that the splicing variant introduces a premature termination codon, which is predicted to result in a truncated protein shorter by 615 amino acids (p.Val2786Argfs*34), and the deletion leads to transcription of a shortened RNA isoform. We suggest that these variants are hypomorphic and partially retain protein function, which explains the clinical picture in the patient. In aggregate, our findings provide evidence that rare biallelic recessive variants in *UTRN* cause a novel autosomal recessive multiple congenital arthrogryposis.

## 1 Introduction

Arthrogryposis multiplex congenita (AMC) is a group of genetically heterogeneous diseases characterized by congenital contractures of two or more joints due to fetal hypokinesia (Hall, 2014). AMC encompasses diseases caused by mutations in genes critical for the formation or functioning of skeletal muscles, neuromuscular junctions, motor neurons of the spinal cord, peripheral nerves, connective tissue of tendons and joints, or the central nervous system (Beecroft et al., 2018; Kiefer and Hall, 2019; Dieterich et al., 2019).

Currently, over 450 genes have been described, pathogenic variants in which lead to AMC-associated diseases (Kiefer and Hall, 2019; Hall et al., 2019; Pehlivan et al., 2019; Laquerriere et al., 2022; Turgut et al., 2024; Turgut et al., 2022). Kiefer and Hall. (2019) proposed classification by the functions of the protein products of the genes responsible for their occurrence (Kiefer and Hall, 2019; Hall et al., 2019). To date, in at least 40% of patients, the AMC is shown to be caused by an impairment of the structure and function of proteins that perform muscle contraction in the intrauterine period, i.e., heavy chains of types 3 and 8 fetal myosin, troponins, tropomyosin, myosin-binding protein C1 and titin, as well as enzymes of the metalloprotease family (Hall et al., 2019; Laquerriere et al., 2022).

Here we present the clinical and genetic characteristics of a patient with a novel type of autosomal recessive arthrogryposis caused by newly identified pathogenic variants of the nucleotide sequence in the *UTRN* gene. We believe that the hypomorphic nature of these variants allowed the patient to survive beyond the embryonic stage and explains the specific features of the clinical presentation.

## 2 Materials and methods

Our manuscript presents the clinical and genetic characteristics of a 7-year-old male patient diagnosed with *UTRN*-associated autosomal recessive arthrogryposis multiplex congenita. Written informed consents were obtained from proband’s parents. The study was performed in accordance with the Declaration of Helsinki and approved by the Institutional Review Board of the Research Centre for Medical Genetics, Russia (Protocol No. 2021-3 dated 12 March 2021).

The diagnostic workup included clinical examination, brain MRI, hip joints, hands, feet and long bones of the extremities radiographs. Whole-genome sequencing (WGS) in a trio format, analysis of mRNA, targeted next-generation sequencing of RT-PCR product, Sanger sequencing, Western blot analysis and immunocytochemistry were performed.

### 2.1 Whole-genome sequencing

Blood samples from proband and unaffected parents were collected and WGS was performed using a DNBSEQ-G400 instrument in pair-ended mode (2 × 150 b. p.) with an average on-target coverage of 30× with MGIEasy FS PCR-Free DNA Library Prep Set (BGI, Beijing, China) for library preparation. Bioinformatic analysis was performed using an in-house software pipeline as described earlier with modifications (Marakhonov et al., 2018). In brief, it included quality control of raw reads (FastQC tool v.0.11.5), followed by read mapping to the hg19 human genome assembly (minimap2 v.2.24-r1122), the sorting of the alignments, and the marking of duplicates (Picard Toolkit v.2.18.14). Base recalibration and variant calling were performed using GATK3.8. Variant annotation was performed using the Annovar tool (v.2018Apr16). CNV and SV analysis was performed using the Manta tool (v.1.6.0). Further filtering was performed through functional consequences and population frequencies, according to the ACMG recommendations as well as clinical relevance determined by the Human Phenotype Ontology database.

Quality Metrics: Proband: The average coverage for the sample was 37×, with 97.89% of the genome covered at ≥10×. Total reads: 323,685,174. Total variants identified: 5,159,810.

Mother: The average coverage for the sample was 38×, with 97.94% of the genome covered at ≥10×. Total reads: 335,772,788. Total variants identified: 5,219,867.

Father: The average coverage for the sample was 32×, with 96.95% of the genome covered at ≥10×. Total reads: 277,796,228. Total variants identified: 5,083,194.

In proband a total of 5,159,810 variants were identified, with 856,075 passing the initial filter. Variant filtering was based on frequency (less than 1% in gnomAD v.3.1.2) and coding sequence effect, including missense, nonsense, coding indels, and splice-site variants. Bioinformatics tools - BayesDel_addAF, DANN, DEOGEN2, EIGEN, FATHMM-MKL, LIST-S2, M-CAP, MutationTaster, SIFT, NetGene2, and SpliceAI - were used to predict the potential impact of the identified variants on protein function. The clinical significance of the variants was evaluated according to guidelines for massive parallel sequencing (MPS) data interpretation. This resulted in 434 candidate variants. Among these, one variant was located in *FLNB* gene (NM_001457.4:c.4861 + 4A>G). The remaining 433 variants were analyzed to rule out other possible causes of the disease. Within this group, two variants (splice site variant (NМ_007124.3:c.8434 + 1G>A) and large deletion spanning exons 3–51) were found in the *UTRN* gene. However, this gene has not been previously associated with any disease in the OMIM database. Only one publication has reported a patient with arthrogryposis carrying an extended duplication that encompasses the *UTRN* gene (Tabet et al., 2010).

### 2.2 Analysis of mRNA

Analysis of mRNA structure was performed using RNA extracted from fibroblasts of the proband and both parents. All cell cultures were obtained and deposited in the Moscow Branch of the Biobank “All-Russian Collection of Biological Samples of Hereditary Diseases. Total RNA was isolated using the Extract RNA reagent (Evrogen, Russia). Reverse transcription was performed with oligo-dT primers using the Reverse Transcription System (Dialat, Russia), in accordance with the manufacturer’s protocol. The quality of the obtained cDNA was assessed by performing qPCR of housekeeping genes. To assay the influence of the variant NМ_007124.3:c.8434 + 1G>A on mRNA structure, the target locus of *UTRN* was amplified using the following primers: 5′- GCA​GGG​AGC​TAT​GGA​TGA​CC-3’ (exon 55) and 5′- TGC​TGT​ACG​GTA​GGC​AGA​AA-3’ (exon 60) with further next-generation sequencing.

To evaluate the impact of the variant NM_001457.4:c.4861 + 4A>G on mRNA structure, the *FLNB* target region was amplified using the primers 5′-CAA​TGG​TGA​TGG​GAC​CTG​CT-3’ (exon 2) and 5′-GAA​AGT​CCG​GCC​TTC​TGA​CA-3’ (exon 53), followed by NGS.

### 2.3 Targeted next-generation sequencing of RT-PCR product

NGS libraries were prepared using the “SG GM” Kit (Raissol) and sequenced on the FastaSeq 300 High-throughput Sequencing Platform (GeneMind, China) in a paired-end mode (2 × 150 b. p.). The targeted locus had a coverage over 300′000x in the proband’s samples and over 150000x in the proband’s parents samples. The raw sequencing data was processed with a custom pipeline based on open-source bioinformatics tools. In brief, the pipeline involved quality control of raw reads using the FastQC tool v0.12.1, followed by read mapping to the hg19 human genome assembly and sorting using STAR 2.7.11b. Splice junctions, such as exon skipping, were visualized using Sashimi plots in IGV.

### 2.4 Sanger sequencing

Validation of the deletion NC_000006.11:g.144713636_144923588del identified through WGS was performed using Sanger sequencing. cDNA samples from the proband’s and his parents’ fibroblasts were used as templates. The PCR was conducted using the ProFlex PCR System (Applied Biosystems). Visualization of the obtained PCR products was carried out using agarose gel electrophoresis. The proband’s PCR product corresponding to the deletion allele was gel-extracted, purified (Evrogen, Russia), and submitted for Sanger sequencing (performed in-house by Evrogen). The following primers were used 5′-AGC​TGA​ACC​ATC​GTA​GGA​AGT-3’ (exon 21) and 5′-GGA​GCT​GTA​AGG​CTG​GAA​CA-3’ (exon 36).

### 2.5 Western blot analysis

Primary dermal fibroblasts were seeded on Petri dishes and cultivated for 24 h before cell homogenates preparation. Cells were washed with PBS, briefly washed with water and homogenized in lysis buffer (200 mM Tris-HCl pH = 6.8, 4% SDS, 25% glycerol, 1 M β-mercaptoethanol, complete protease inhibitor cocktail (p8340, Sigma-Aldrich) and heated at 95 °C for 5 min. Lysates were subjected to 6% SDS-polyacrylamide gel electrophoresis and proteins were transferred into a PVDF membrane (BioRad). Then 5% skim milk in TBST was used for blocking the membrane and for antibody incubation. For staining primary rabbit polyclonal anti-utrophin antibodies (Abcam Cat# ab244363, RRID:AB_3678559) and secondary anti-rabbit igG, HRP-linked antibodies (Cell Signaling Technology Cat# 7074, RRID:AB_2099233). After washout membranes were subjected to enhanced chemiluminescence detection analysis using SuperSignal™ West Femto Maximum Sensitivity Substrate (ThermoFisher Scientific).

### 2.6 Immunocytochemistry

Fibroblasts from patient, parents and control individuals were grown on coverslips and fixed and permeabilized in methanol, washed in PBS and incubated with incubated with primary anti-utrophin antibodies (Abcam Cat# ab244363, RRID:AB_3678559) and secondary Alexa Fluor™ 488-conjugated anti-rabbit-IgG secondary antibodies (Molecular Probes Cat# A-11008, RRID:AB_143165) and DAPI. Images were collected by Leica STELLARIS 5 microscope (x60, oil immersion) from each sample. Image acquisition parameters (gain on CCD, excitation intensity, exposure time) were same for all samples.

## 3 Results

### 3.1 Clinical findings

**FIGURE 1:**
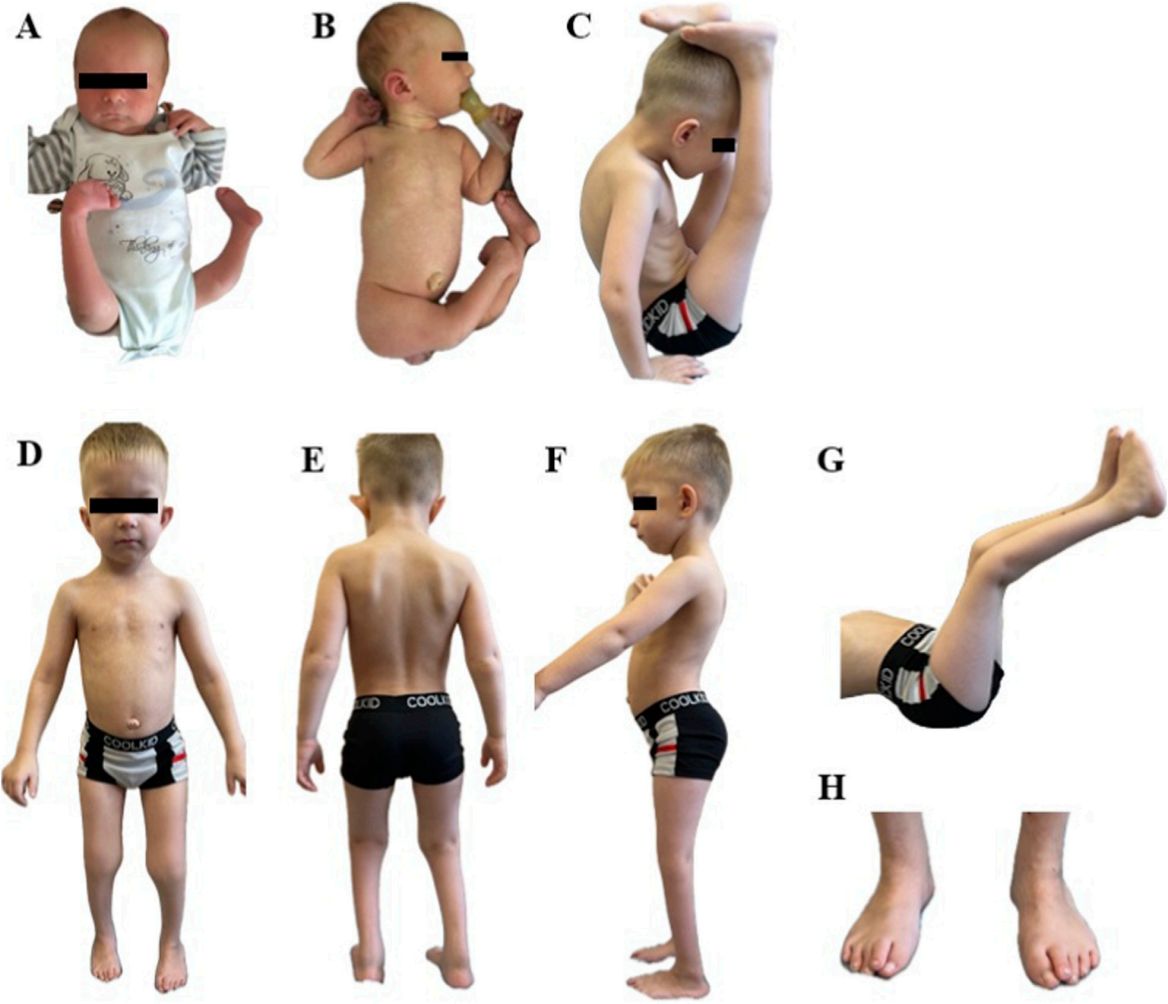
**(A,B)** phenotype at birth; **(C–H)** phenotype at the age of 7 years.

The proband is a 7-year-old boy, the first child of healthy non-consanguineous parents of Russian origin. Starting from the 19th week of gestation fetal ultrasound examination showed bilateral equinovarus foot and delayed fetal development. After the 25th week reduced fetal movements, oligohydramnios, fetal growth restriction were found. The child was born from induced operative labor at the gestational age of 40 weeks with a weight of 2,890 g (−1.07 SDS), a length of 54 cm (1.46 SDS) and an Apgar score - 4 point at 1′, 7 points at 5’; due to meconium aspiration, resuscitation and respiratory support were required for several minutes. From birth, there were right nasolacrimal duct obstruction, umbilical hernia, as well as bilateral hip dysplasia, extensor contractures of the knee joints, bilateral equinovarus foot, flexor contractures in the elbow joints; thus, AMC was suspected (Figures 1A,B). Early motor development was delayed: he began to hold his head from 5 months, to walk from 4 years old. From the age of two, surgical interventions were performed to treat equinovarus foot; subsequently, orthopedists determined the right dystrophic coxa vara, shortening of the right lower limb by 1.5–2 cm. From the age of three, a significant growth delay was noted; on the radiography of the hand, performed at 3 years and 4 months, the bone age corresponded to 2 years. Up to 3 years of his life, he had difficulty chewing solid food, and up to 5 years he had constipation due to megadolichosigma shown by irrigoscopy. Psycho-speech development was delayed; from the age of 3 years and 9 months, phrasal speech with tonic-clonic stuttering appeared. There were no seizures and epileptiform activity.

According to the examination at the age of 7 years, height was 101 cm (−3.88 SD), body weight was 13.7 kg (−3.6 SD), and head circumference was 50.5 cm (−1.13 SD). Dysmorphic facial features included a large forehead with a prominent metopic suture, hypertelorism, a wide nose, a narrow almond-shaped eyes, swollen eyelids, partial right ptosis, thin lips, micrognathia, high palate, large cup-shaped protruding low-lying ears. An additional nipple was found on the left; also, he had an umbilical hernia, skin dimples in the area of the elbow joints and popliteal areas, transverse palmar creases. Examination also showed cone-shaped fingers, hypermobility of the distal phalanges, the leading middle finger on the right hand, as well as narrowing of the feet, inward-oriented great toe with overlapping second toe, protruding heels and lumbar hyperlordosis. Limited external hip rotation, extensor contractures of the knee joints with overextension, shortening of the right lower limb, gait disturbance due to contractures of the ankle joints and foot deformities were also observed. According to the neurological examination the child also had diffuse hypotonia, hyporeflexia, hypotrophy (mainly the muscles of the lower legs), weakness of the neck flexors and abdominal muscles (3/5 on the Medical Research Council scale) (Figures 1C–H). CK level measured at 57 U/l fell within the normal range.

Brain MRI demonstrated nodular heterotopia of subependimar and subcortical gray matter of both cerebral hemispheres; in the right hemisphere, a ribbon-shaped heterotopia deforming the contour of the lateral ventricle and a cyst of the left lateral ventricle were found (Supplementary Figures 1A,B). Radiographs of the hip and knees joints are presented in Supplementary Figures 1C–H.

### 3.2 Molecular findings

**FIGURE 2:**
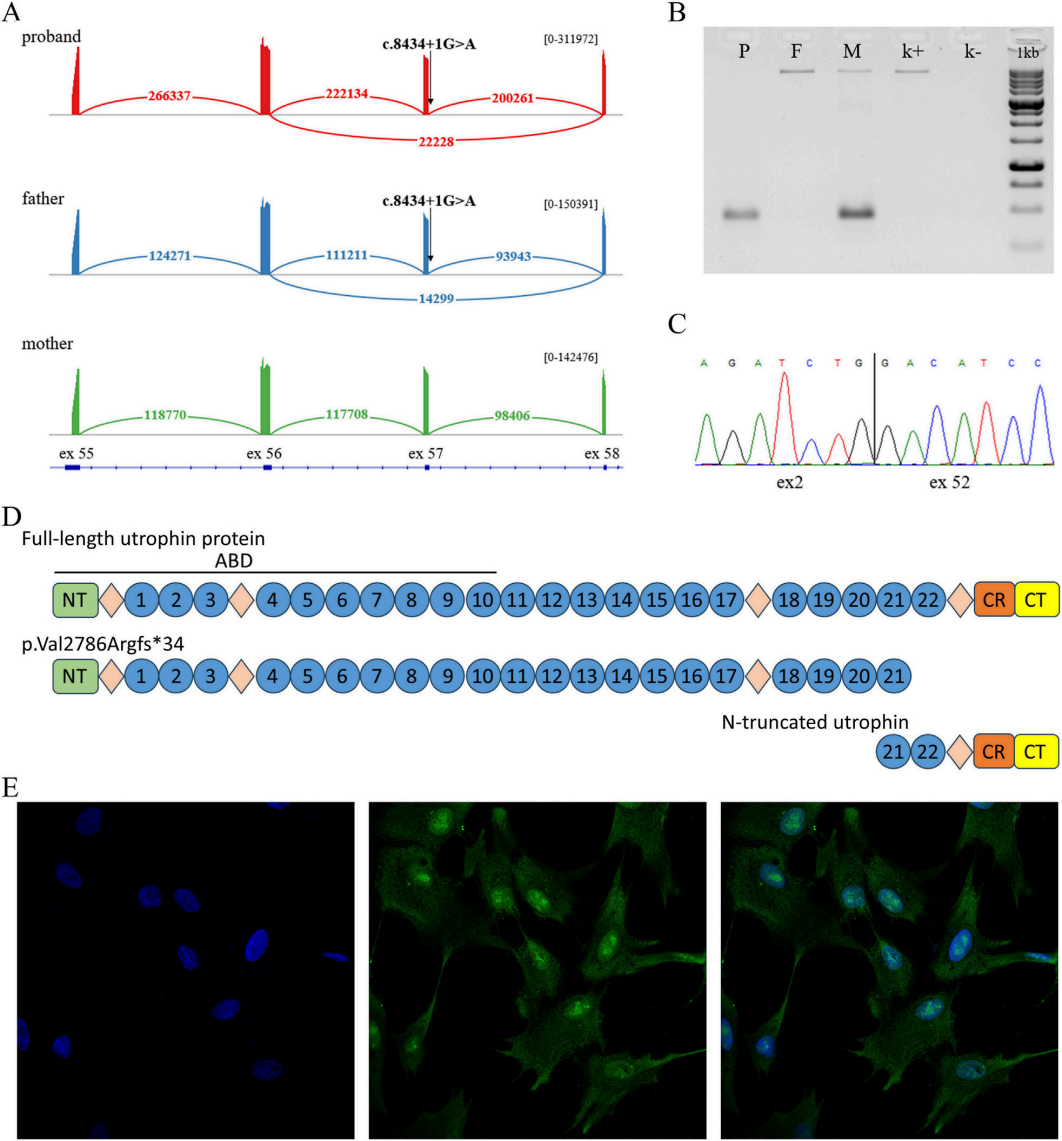
**(A)** Sashimi plot with exon-exon junction visualization of deep sequencing of RT-PCR products in a proband sample, heterozygous carrier of the c.8434 + 1G>A variant (red), a proband’s father, heterozygous carrier of the c.8434 + 1G>A variant (blue), a proband’s mother (green). Numbers indicate the amount of exon-exon junction reads for each isoform. **(B)** Agarose gel electropheresis of RT-PCR products containing exons 2-53 of the UTRN gene. Lower bands correspond to the presence of the deletion, upper bands correspond to wild type (patient (P), parents (M, F) and healthy control (k+), NTC (k-)). **(C)** Validation of the deletion identified in the proband through WGS using Sanger sequencing. **(D)** Schematic of full-length utrophin and the patient’s truncated protein variants (ABD–actin-binding domain; NT–N terminus; circles–spectrin-like repeats; diamonds–disordered hinge regions; CR–cysteine-rich domain; CT–C terminus). **(E)** Immunocytochemistry of proband fibroblasts (DAPI, UTRN, MERGE).

WGS in a trio format revealed variant NМ_007124.3:c.8434 + 1G>A in the canonical donor splice site of intron 57 of the *UTRN* gene in the proband and healthy father. To assess the impact of the c.8434 + 1G>A variant on mRNA splicing, a bioinformatics analysis was conducted using the splice prediction program SpliceAI. The analysis showed that the c.8434 + 1G>A variant leads to disruption of the canonical splice donor site, which can lead to exon skipping. To verify this prediction, we performed RNA analysis on the *UTRN* gene locus containing the splice variant. To do this, we isolated RNA samples from the fibroblasts of the proband and his parents and performed RT-PCR followed by deep targeted sequencing (Figure 2A).

In addition to the reference mRNA isoform, we found an isoform with the skipping of exon 57, which was absent in the mother and in the control sample. Based on this data and the bioinformatics analysis, it can be concluded that the c.8434 + 1G>A variant leads to exon skipping. At the protein level, this event results in the shortening of the protein by 615 amino acids (p.Val2786Argfs*34).

Sequence analysis of the proband’s locus also revealed a common heterozygous, likely benign variant chr6:145093052A>G (rs80110270). Interestingly, this variant was in a state of strong allelic imbalance (WT/Mut = 10/90). This data suggests a significant reduction in the expression of the allele containing the c.8434 + 1G>A variant, which may be a result of transcript degradation via the NMD mechanism.

Furthermore, WGS analysis revealed a deletion NC_000006.11:144713636_144923588del, encompassing exons 3 to 51 and the flanking intronic regions. The presence of the deletion was confirmed using RNA analysis. cDNA was used as the template, since cDNA sequence analysis allows for the confirmation of the variant’s presence and the assessment of the relative expression levels of the alleles. The sequence analysis confirmed the presence of the deletion in both the proband and the proband’s mother and showed that the deletion does not lead to a reduction in the expression of the allele containing it (Figures 2B,C; Supplementary Figure 2).

To evaluate whether the proband retains functional UTRN protein, we analyzed fibroblasts from the patient, both parents, and healthy controls using immunocytochemistry (ICC). The results confirmed that the proband expresses utrophin (Figure 2D), with no apparent differences in fluorescence intensity observed between the proband, his parents, and control samples (Figure 2E; Supplementary Figures 3). Additionally, we were able to detect, both, the wild-type and the C-terminal truncated proteins in paternal fibroblasts (Supplementary Figures 4). However, the N-terminal truncated protein (∼100 kDa) was undetectable because the deletion removes the epitope recognized by the antibody.

WGS data analysis additionally identified the variant NM_001457.4:c.4861 + 4A>G in the *FLNB* gene. This variant was absent from population databases (gnomAD v2.1.1) and segregation analysis confirmed its inheritance from the healthy father. Based on population data and segregation findings, in accordance with ACMG criteria (PM2, BS4), the variant was initially classified as one of uncertain clinical significance (VUS). Pathogenic variants in the *FLNB* gene are associated with Larsen syndrome (autosomal-dominant inheritance), primarily with missense variants rather than loss-of-function (LoF) variants. Regarding the clinical presentation, Larsen syndrome is associated with multiple dislocations secondary to generalized joint laxity, distinctive facial features and characteristic ossification pattern of calcanei on the radiographs (accessory calcaneal ossification center) (Girisha et al., 2016).

To further assess the pathogenicity of the c.4861 + 4A>G variant, we performed mRNA structure analysis of the *FLNB* gene using targeted deep sequencing. Sashimi plot visualization of the sequencing data revealed no detectable impact of the variant on mRNA splicing (Supplementary Figures 5). Given that the proband’s clinical and radiological findings demonstrate opposite features (joint contractures, other facial appearance, normal ossification pattern) and his father shows no signs of skeletal combined with RNA analysis demonstrating no aberrant splicing effects, we conclude that the *FLNB* c.4861 + 4A>G variant is unrelated to the observed phenotype. Based on these findings and in accordance with ACMG guidelines (PM2, BS4, BS3), this variant should be classified as benign.

## 4 Discussion

It is known that utrophin is an autosomal homologue and a fetal form of dystrophin, since its expression level reaches a maximum on day 13.5 of the embryonic period and then decreases rapidly, while dystrophin is expressed mainly in the postnatal period. Utrophin and dystrophin have a similar protein structure and, according to their main function, are building proteins that stabilize the state of the membrane of muscle cells (Blake et al., 1996; Szwec et al., 2024). The *DMD* and *UTRN* genes are among the longest protein-coding genes detected in the genome. It was expected that pathogenic variants in the *UTRN* gene would be as common as in *DMD*, however, to date, no hereditary monogenic disease with pathogenic variants in the *UTRN* gene has been described in the literature. Given the absence of reported cases, a number of authors believed that the disease for which this gene would be responsible should be relatively rare or has autosomal recessive inheritance (Blake et al., 1996). Another explanation that exists may be a specific expression of the *UTRN* gene, mainly during embryonic development, which may indicate that pathogenic variants in this gene can lead to perinatal fatal disease. In the gnomAD v4.0 population database, only one homozygous variant NМ_007124.3:c.5907-1G>A is found in 4 subjects, which leads to the destruction of the acceptor splicing site of 42 exons of the *UTRN* gene. According to the predictions of SpliceAI, this leads to the deletion of 4 amino acids, which does not lead to a significant change in the structure and possibly does not affect the protein function, which explains the presence of a homozygous variant in healthy people.

The *UTRN* gene is localized on the long arm of chromosome 6, and multiple cases of 6q duplication syndrome are reported, characterized by a specific phenotype: moderate or severe mental retardation, microcephaly, growth retardation, hypertelorism, downslanting palpebral fissures, sunken upper lip, short neck (Tabet et al., 2010; Zweier et al., 2008; Cappon et al., 2000; Grati et al., 2005). Most of the described patients also had congenital arthrogryposis and moderate skeletal abnormalities. Due to the fact that in most patients there were no clear boundaries of duplication, analyzing the contribution of individual genes included in the duplication to the formation of the disease phenotype was challenging. Only Tabet et al., (2010) described an 8-year-old girl with 13 Mb *de novo* duplication in the region of chromosome 6q24.2-q25.3, presenting with mild intellectual deficiency, dysmorphic features, lower leg muscle atrophy, knee and ankle joints stiffness. Using RT-PCR and several intragenic and intergenic probes, the authors showed the proximal breakpoint between exons 42 and 54 of the *UTRN* gene. Since utrophin is a skeletal muscle structural protein predominantly localized in musculoskeletal joints, the authors proposed that its dysfunction underlies the contractures and muscle atrophy (Tabet et al., 2010). Notably, the patient’s phenotype, including congenital contractures, closely overlaps with other reported 6q23-q26 duplication cases (Cappon et al., 2000; Grati et al., 2005).

Despite the clear molecular interpretation of the pathogenicity of the detected deletion and splicing variant, their damaging impact on protein function may not be so straightforward. Utrophin is a large structural protein, and, similar to dystrophin, there are peculiarities in interpreting various molecular events including those leading to partial loss of function. For example, we demonstrated that the splicing variant c.8434 + 1G>A results in a protein shortened by 615 amino acids (out of 3,433). However, the large N-terminal portion of the protein may still be translated and partially retain its function. Such cases have been reported for the *DMD* gene, where a 2-bp deletion in exon 74 of *DMD* was found in a patient with Becker muscular dystrophy. According to immunohistochemical analysis, the patient’s dystrophin levels were lower than in control samples but were sufficient to cause Becker muscular dystrophy rather than the more severe Duchenne phenotype (Kimura et al., 2009). The second pathogenic variant identified in the patient, a deletion spanning exons 3 to 51, leads to the transcription of a shortened RNA isoform. This RNA could still be translated into utrophin protein from Kozak sequences located in exon 52, potentially producing a truncated protein lacking the N-terminal portion but retaining a substantial C-terminal region. Our immunohistochemical analysis revealed utrophin expression in the patient’s fibroblasts, suggesting that despite the truncation, the protein is expressed at levels comparable to those in parental and control samples. Thus, we suggest that both variants may exhibit a hypomorphic effect, where part of the gene’s function is preserved, which could explain the lack of an embryonic lethal effect.

## 5 Conclusion

Thus, we present for the first time a patient with two hypomorphic pathogenic nucleotide variants in a compound heterozygous state in the *UTRN* gene. Clinical manifestations of the disease were characterized by a combination of AMC, short stature, hypotrophy of the lower leg muscles, dysmorphic facial features and brain, colon, lacrimal canal abnormalities, which may be due to the ubiquitous expression of utrophin in the embryonic period.

## Data Availability

The data generated and analyzed in this study are not publicly available due to ethical restrictions and institutional policies regarding patient confidentiality. The informed consent obtained from participants does not allow for public data sharing, in compliance with the approved protocol by the ethics committee. However, the data may be made available to qualified researchers upon reasonable request and with appropriate ethical approvals. The original contributions presented in the study can be found here: https://www.ebi.ac.uk/ena/browser/view/PRJEB101133 with accession number ERP182562.
