# Supplementary material for: Biallelic variants in the UTRN gene cause a novel form of multiple congenital arthrogryposis
Source: Front Genet. 2025 Oct 30;16:1664424. doi: 10.3389/fgene.2025.1664424 (PMC12611563; doi:10.3389/fgene.2025.1664424)
Supplement: Supplementary file 1 [file Supplementaryfile1.docx]

Supplementary Material

# Supplementary Figures


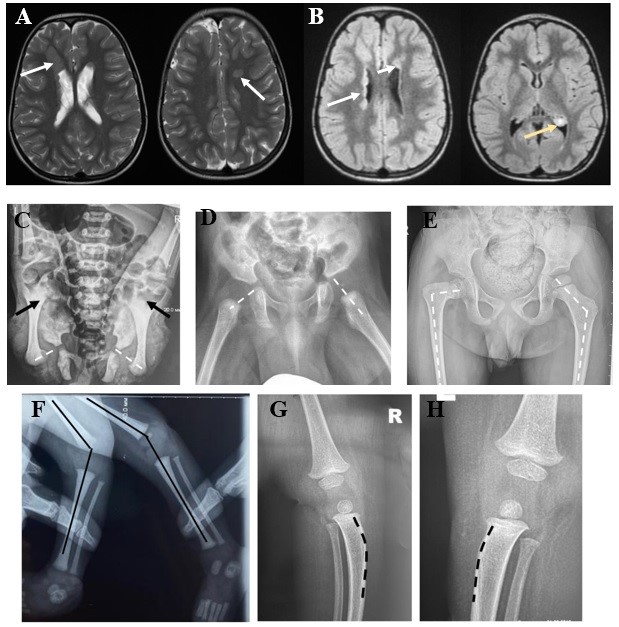


**Supplementary Figure 1.** Brain magnetic resonance imaging at the age of 7 years: A - T2-weighted image, B – Fluid attenuation inversion recovery (FLAIR) image: nodular heterotopia of subependimar and subcortical gray matter of both cerebral hemispheres (white arrows); in the right hemisphere, a ribbon-shaped heterotopia deforming the contour of the lateral ventricle; cyst of the left lateral ventricle (yellow arrow); C-E - Anteroposterior radiographs of the hips: C – at birth: abnormal position with the extreme flexion of the hips (black arrows), properly centered hip joints (white broken lines), D - at 1 year: delayed ossification of the femoral heads, properly centered hip joints (white broken lines), E - at 6 years: normal parameters, left side; coxa vara, right side (abnormal neck-shaft angle – 100 degrees (white broken lines); F-H - Lateral radiographs of the knees: F - at birth: hyperextension of the both knee joints (black lines), G-H - at 6 years: antecurvatum deformity of proximal tibia (white broken lines).


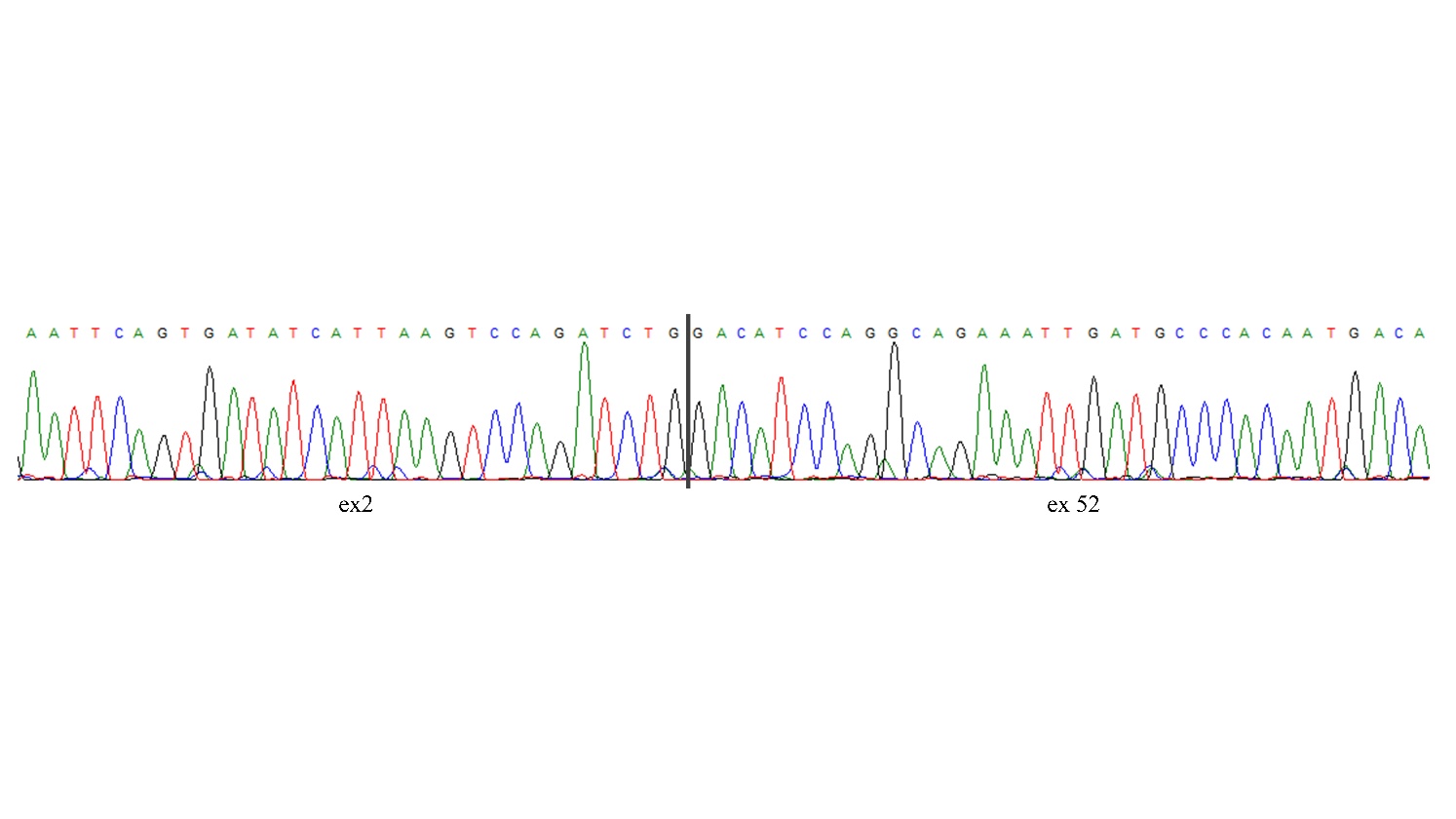


**Supplementary Figure 2.** Sanger sequencing chromatogram confirming the skipping of exons 3 to 51 in the proband's cDNA.


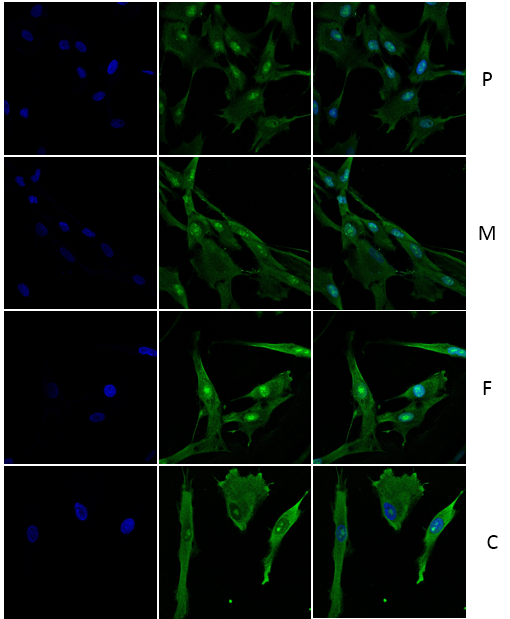


**Supplementary Figure 3.** Immunocytochemistry analysis of fibroblast from the patient (P), parents (mother (M) and father (F)) and healthy control (С), stained for DAPI, UTRN, and merged channels.


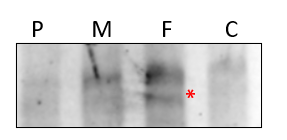


**Supplementary Figure 4.** UTRN western blot analysis from dermal fibroblasts. Shorter isoform is marked with red star.


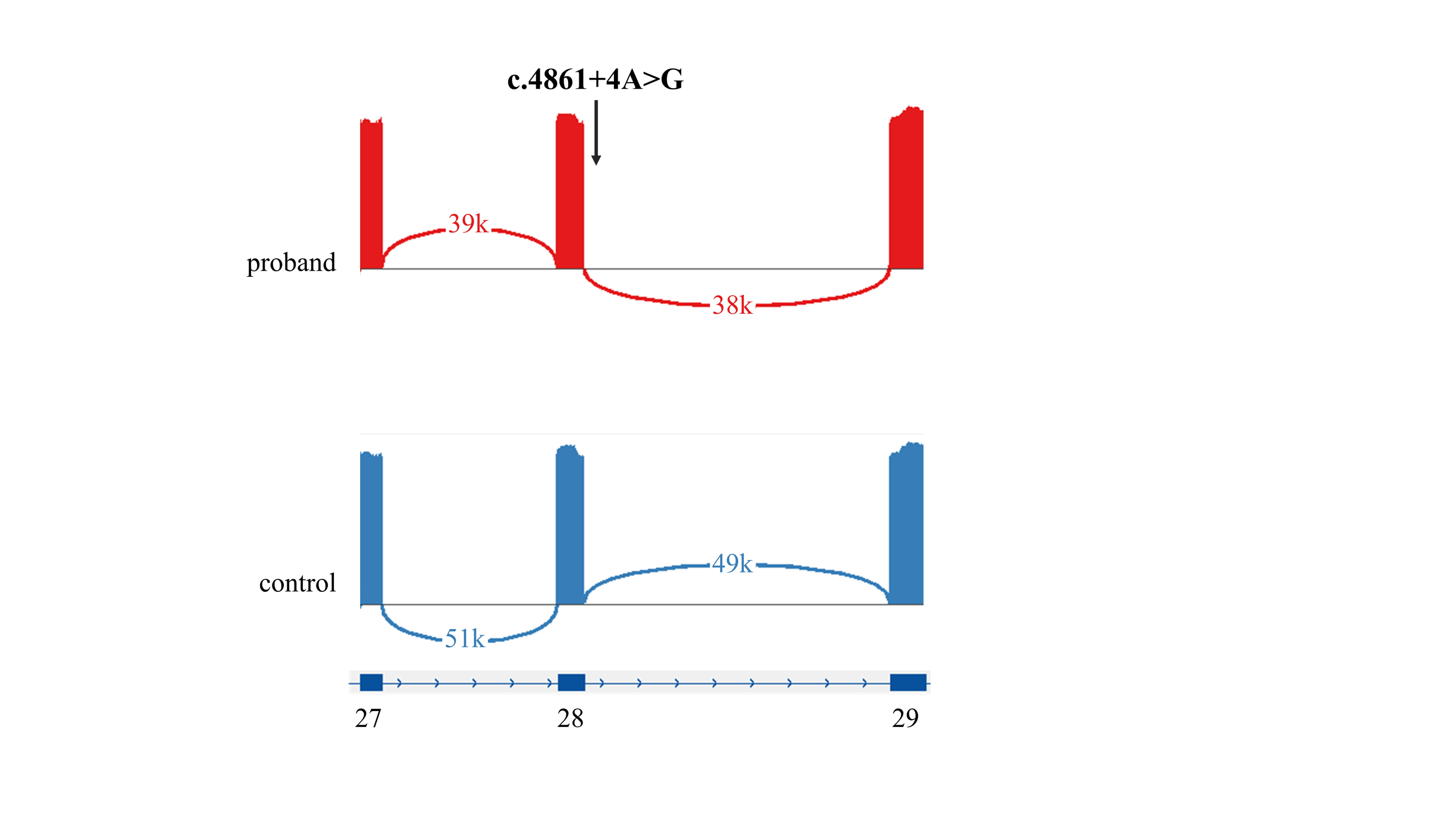


**Supplementary Figure 5.** Sashimi plot with exon-exon junction visualization of deep sequencing of RT-PCR products in a proband sample, heterozygous carrier of the c.4861+4A>G in the *FLNB* gene variant (red), and control sample (blue).
